# An investigation of whether factors associated with short-term attrition change or persist over ten years: data from the Medical Research Council Cognitive Function and Ageing Study (MRC CFAS)

**DOI:** 10.1186/1471-2458-6-185

**Published:** 2006-07-18

**Authors:** Fiona E Matthews, Mark Chatfield, Carol Brayne, Medical Research Council Cognitive Function and Ageing Study (MRC CFAS)

**Affiliations:** 1 MRC Biostatistics Unit, Institute of Public Health, Robinson Way, Cambridge, CB2 2SR, UK; 2 Department of Public Health and Primary Care, University of Cambridge Institute of Public Health, Robinson Way, Cambridge, CB2 2SR, UK; 3 MRC Human Nutrition Research, Elsie Widdowson Laboratory, Fulbourn Road, Cambridge, CB1 9NL, UK

## Abstract

**Background:**

Factors associated with the loss of participants in long-term longitudinal studies of ageing, due to refusal or moves, have been discussed less than those with short term follow-up.

**Methods:**

In a population-based study of cognition and ageing (the Medical Research Council Cognitive Function and Ageing Study (MRC CFAS)), factors associated with dropout due to refusal and moving in the first follow-up period (over two years) are compared with factors associated with dropout over ten years. Participants at 10-year follow-up are compared with their age-standardised baseline contemporaries.

**Results:**

Some consistent trends are found over the longer term. Refusers tended to have poorer cognition, less years of education, not have a family history of dementia and be women. Characteristics of people who moved differed between waves, but the oldest and people in worse health moved more. When surviving and responding individuals at ten years are compared with those of the same age at baseline many differences are found. Individuals of lower social class, education, cognitive ability, in residential care, with sight/hearing problems and poor/fair self-reported health are less likely to be seen after 10 years of follow-up. Individuals report more health problems when they participate in multiple interviews.

**Conclusion:**

The characteristics of refusers in the longer term are similar to those refusing to participate over the shorter term. Long-term follow-up studies will under represent the disadvantaged and disabled but represent full health status of participating individuals better. There are advantages and disadvantages to both short-term and long-term follow-up.

## Background

Dropout or attrition, not due to death, in a longitudinal study can present a threat to the validity of population based longitudinal data analysis and is considered unnatural attrition. Assessment of the health of a population requires information on the total population, but individuals who do not respond to health surveys are not a random subset of those seen. Over the long term a study sample will be subject to mortality, however it is essential to investigate individuals who refuse to participate in new interviews as they may have differing health needs. Many longitudinal studies have short follow-up periods, but increasingly studies are now undertaking multiple waves of interviews over many years and may have differential attrition.

A recent systematic review of the attrition between waves in large population based studies of older people noted that, although most report dropout, few publish an analysis of factors associated with refusal[1]. In those where multivariable analysis has been undertaken increasing age and poor cognition are the only factors consistently associated with refusal, whilst individuals who change residence tend to be older and have poorer health[1].

Several large studies on ageing have analysed dropout over multiple waves or beyond five years of follow-up [2-5]. Analyses reported to date concentrate on repeated analysis of differences between baseline to the latest wave rather than changes between waves. These analyses are therefore not independent, yet report on factors changing over time by changing significance and strength of association[2,3,6]. The same studies have the potential to address whether factors associated with attrition are persistent or change with increasing number of waves and follow-up time. Others have calculated attrition effects between multiple waves investigating a common set of factors associated with attrition[4,5]. Only one small study investigated differentials with low verbal IQ associated with attrition at four years and less education at eight years[7].

The Medical Research Council Cognitive Function and Ageing Study (MRC CFAS) is a large population based study that began in the early 1990s[8]. Individuals were more likely to refuse at two-year follow-up if they had poor cognition, less years of education, lived in their own home, did not live alone, and lived in a rural region at baseline[9]. Individuals were more likely to move from the study areas if they were very old, were not married, ever smoked, ever had self-reported depression, or unable to complete the Mini-Mental State Examination (MMSE)[10] or had symptoms of dementia[9]. Individuals who lived in warden controlled accommodation moved less[9].

A recent wave of interviews (at year 10) has been completed. This paper addresses questions on whether risk factors for refusal and moving are consistent between the later waves or whether new factors emerge over time. Dropout due to death is not a focus of this paper as previous MRC CFAS analyses have examined mortality over 2 years [9], and over 5 years[11], reporting that dropout due to death is associated with poor cognition, increasing age, functional impairment, poor self-reported health, ever smoked and being male.

This paper aims to address two questions.

• Are factors previously seen as important for dropout (refusers and movers) at two-year follow-up also related to subsequent dropout?

• Are there systematic differences between responders at year 10 and those who were the same age at the baseline interview?

The overall aim is to investigate whether prolonged follow-up introduces potential biases beyond age effects.

## Methods

MRC CFAS is a population based longitudinal study of health in the older population [12]. The initial phases of the study have been described in detail elsewhere[8,13,14]. This paper focuses on five centres in England and Wales (East Cambridgeshire, Gwynedd, Newcastle-upon-Tyne, Nottingham and Oxford) with ten years of follow-up. All centres except Gwynedd obtained the population information from the appropriate Family Health Service Authorities (FHSA) list; all individuals aged 64 and over on defined dates were enumerated. Population-based samples stratified to ages 65–74 years and 75 and above were taken to achieve approximately 2,500 interviews at each centre. In Gwynedd enumeration was undertaken by searching records in general practioners' surgeries. All individuals have been flagged at the Office of National Statistics (ONS) for deaths and emigrations.

**Figure 1:**
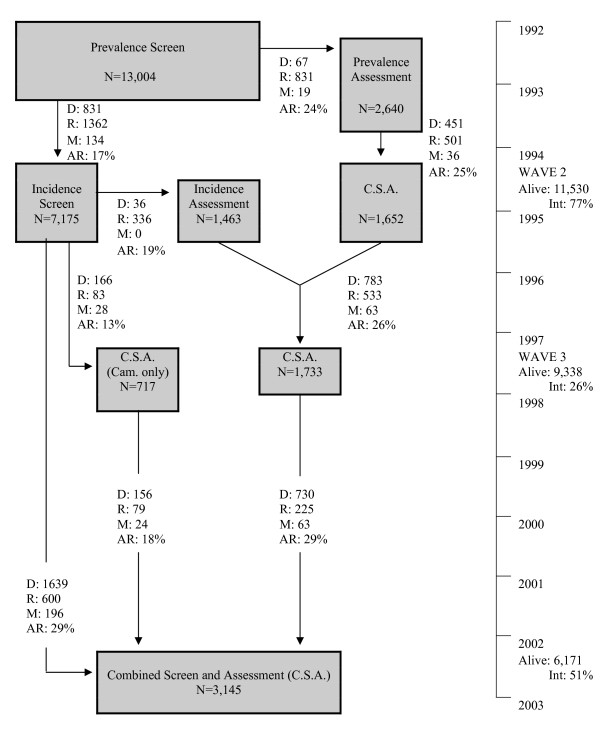
**MRC CFAS design over ten years with loss to follow-up information**. Key: N number interviewed; D – died between interviews; R – refused to be interviewed; M – moved between interviews; AR – attrition rate i.e. (R + M)/(R + M + N); C.S.A. – Combined Screen and Assessment; Alive – Number of baseline participants alive at median time of wave interviews; Int – Percentage of those interviewed out of those alive. Notes: Data version 8.0. Numbers may be inconsistent between waves due to temporary refusers. Position of boxes corresponds to the interquartile range of the date of the interviews.

The flowchart of the interviewing in MRC CFAS is shown in Figure 1. At wave one a screening interview (defining baseline) was undertaken (non-response rate of 20%), followed a three months (median) later by a more detailed assessment interview on a 20% sub-sample of participants, biased towards the cognitively impaired. Two years later, all participants were re-interviewed (year 2). Individuals who had been assessed were re-assessed by a combined screen and assessment interview. All other individuals were re-screened with a new assessment group selected to be biased to the cognitively impaired following a similar selection processes to that at baseline [14]. Five to six years after baseline (year 6), participants that had previously been assessed were re-assessed (n = 1733). However, extra funding meant that all participants in the Cambridgeshire centre (an extra n = 717) were assessed at this time. Ten years after baseline (year 10), everyone who had not refused an interview, moved or died was re-interviewed (n = 3145).

Structured interviews were undertaken in the respondent's own home using a computerised interview. The screen interview collected information about demographics including marital status and educational ability, social class, cognitive ability (Mini Mental State Examination [10]), functional ability (modified Townsend disability scale[15] and reduced to an activities of daily living-instrumental activities of daily living (ADL-IADL) scale[16], organicity symptoms of the Automated Geriatric Examination for Computer Assisted Taxonomy (AGECAT[17]) providing organicity levels O0–O5, self-reported chronic diseases (including cardiovascular diseases, stroke and the Rose angina questionnaire[18]), emotional problems, smoking history, alcohol consumption, self-perceived health, sensory problems (eyesight and hearing), and diseases of first degree relatives.

At each interview, individuals were classified as undertaking that interview successfully or not, and if not, reasons for non-response were ascertained. Once an individual had refused an interview or moved away they were not contacted again. Individuals could temporarily say the interview was 'not convenient' and these were re-contacted at the next wave. In a small number of cases, those who were unable to undertake the complete interview had some sections completed by proxy. Individuals for whom a complete interview was undertaken by a proxy were not seen again at the next wave.

Individuals who were alive but were not interviewed are described in this paper as 'refusers', unless they moved out of the study areas, or could not be contacted at the present address, in which case they are described as 'movers'. In this analysis we only consider attrition in those who were alive during the interview period. The time of the interview wave is set to be the median of follow-up time to reflect the real interview waves.

MRC CFAS has Multi-centre Research Ethics Committee approval and ethical approval from the relevant Local Research Ethics Committees. Participants gave informed consent. Data from the study have been released in stages. Version 8.0 of the data has been used, and information from ONS for deaths, loss to follow-up and emigrations is complete to the end of December 2004.

### Model fitting

Some factors related to dropout are correlated with each other and not independently associated with dropout. Multivariable logistic regression analyses have been undertaken to investigate potential independent factors associated with dropout. All models satisfied the Hosmer Lemeshow goodness of fit test based on deciles of risk (all p > 0.2). All factors were investigated univariately and if associated with attrition (p < 0.1) were included within the multivariable model. All factors that remained significantly associated with attrition at the 5% level remained in the model. Factors that vary with time were updated at each completed interview.

For refusal, the number of intervening interviews (0, 1 or 2) has been included in the model as it is a confounder in the relationships of refusal to age and MMSE. For completeness the analysis of refusal and moving from baseline to year 2 has been presented with similar methodology to the above.

### Representation of year 10 data

Many factors associated with attrition also change with increasing age. To investigate differences of the population studied at year 10 two methods have been used. Firstly an age-standardised group has been investigated using the same age range from baseline as seen at the 10-year follow-up (age 75+ years). Stratification of the original sample on age means there is a disproportionately high number of people aged 75+ years at baseline, and therefore aged 85+ years at 10-year follow-up. For this reason the percentages have been adjusted for the over sampling of the age for all individuals at both baseline and year 10. Secondly an age-matched sample was selected choosing two age- and centre-matched controls from the baseline interview to each case seen in year 10. Exact age matching was possible for all ages up to 95 years then one group of individuals aged 95 years and above.

## Results

Non-mortality dropout ranges from 13–29% between interviews (Figure 1). There is higher dropout and mortality where targeting cognitive impaired, i.e. at prevalence and incidence assessment interviews and subsequent combined screen and assessment interviews. However an equivalent level of dropout (29%) was seen in the individuals who were seen at baseline and year 2 but were not seen again until year 10 (median of 8 years later). The number of baseline participants alive at the start of each wave, and the percentage interviewed is also given in Figure 1. At year 6 interviews 80% of those approached were seen, however this reflects only 26% of the complete baseline participants. As individuals who have refused or moved away were not re-contacted, attrition over frequent series of interviews can accumulate quickly. MRC CFAS has interviewed 51% of the baseline participants who were known to be alive at 10-year follow-up.

**Figure 2:**
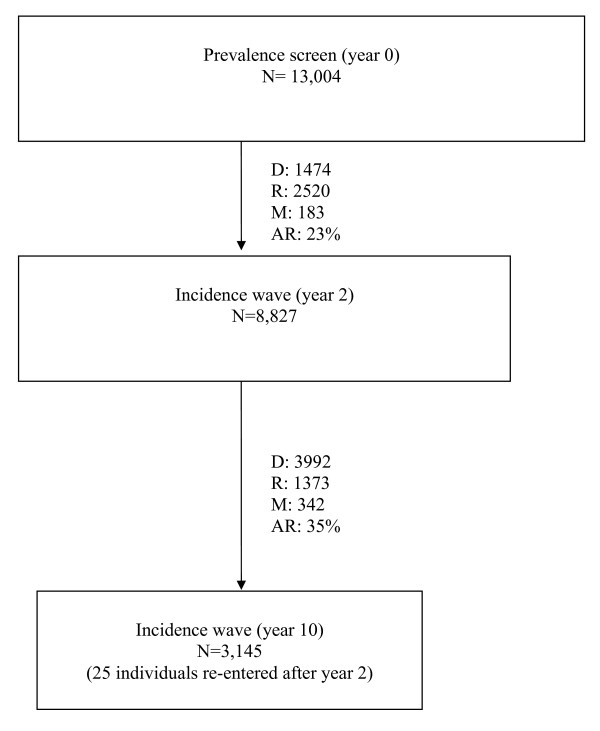
**MRC CFAS major wave information**. Key: N number interviewed; D – died between interviews; R – refused to be interviewed; M – moved between interviews; AR – attrition rate i.e. (R + M)/(R + M + N)

A simplified study diagram is shown in Figure 2. This gives only the information relevant to the analysis presented below.

### Year 2 to year 10 dropout

The characteristics of people seen who participated at year 2 by interview status at year 10 are shown in Table 1. 3992 (45%) of the 8827 individuals interviewed at year 2 had died by year 10 follow-up. Of the 4835 alive, 3120 (64%) were interviewed who had known characteristics at year 2. There were 25 people who were not interviewed at year 2, but were at year 10 who are excluded from this analysis. 1715 (36%) were not interviewed; 342 (19%) had moved, and 1373 (81%) refused. Their characteristics at year 2 are shown in Table 1.

Individuals who completed the incidence screen interview but refused the assessment interview at year 2 are considered to be participants at year 2 (n = 336)[9].

#### (a) Refusers

Individuals who were interviewed at year 2, but refused by year 10 were more likely to be older, women, of manual social class, with no more than statutory (i.e. <=9 years) education, with lower MMSE scores, with screening AGECAT O3 and above, with no family history of dementia, with more disability (both IADL and ADL), with fair/poor self rated health, and were never-smokers (Table 1). In the multivariable analysis a lower MMSE score, poorer self-reported health, increasing age and living in a residential home were strongly related to refusal (Table 2). Refusal was slightly less common in people with a family history of dementia, and slightly more common in those with just statutory education and women. Burden of interviewing adjusts for the extra interview at Cambridge, and the fact that the lower cognitive groups were targeted at some phases (results not shown). The factors affected with refusal at year 10 are very similar to those seen at year 2. Differences are seen with self-perceived health that has a stronger trend at year 10; cognition has a smaller trend at year 10. Undertaking a stratified analysis by age (in three groups) and sex separately showed similar effects of the risk factors within the stratification groups.

#### (b) Movers

Comparing movers with those interviewed in Table 1, movers were disproportionately older, women, with slightly lower MMSE scores. These factors were shown to be the important factors in the multivariable analysis presented in Table 2. The oldest (aged 85+ years at baseline) were twice as likely to move than the youngest (aged 65–74 years at baseline). People with the highest MMSE scores were less likely to move, and women moved more than men. The movers analysis is not as consistent as the refusers analysis with movers at year 2 showing different effects to the movers at year 10, mainly due to relaxation of the rules for multivariable model fitting in the original attrition paper[9]. Originally factors associated with attrition regardless of significance were included in the model due to the small number of individuals; these factors are all shown not to be associated with moving between year 2 and year 10. None of these factors (shown in italics in Table 2) would be considered as being associated with moving. Likewise centre shows a pattern at year 10 which was not there at year 2 and there was no difference between men and women after just two years.

There were some geographical differences for both refusers (χ42
 MathType@MTEF@5@5@+=feaafiart1ev1aaatCvAUfKttLearuWrP9MDH5MBPbIqV92AaeXatLxBI9gBaebbnrfifHhDYfgasaacH8akY=wiFfYdH8Gipec8Eeeu0xXdbba9frFj0=OqFfea0dXdd9vqai=hGuQ8kuc9pgc9s8qqaq=dirpe0xb9q8qiLsFr0=vr0=vr0dc8meaabaqaciaacaGaaeqabaqabeGadaaakeaacqaHhpWydaqhaaWcbaGaeGinaqdabaGaeGOmaidaaaaa@3078@ p = <0.0001 for year 2 and for year 10) and movers (p = 0.56 for year 2 and p = 0.0001 for year 10); no one centre was different to another after adjusting for multiple comparisons. The patterns seen in the centres were not consistent between year 2 and year 10 indicating differences may be chance findings.

### Characteristics of participants at year 10 compared to individuals of the same age baseline

Table 3 shows the characteristics for individuals at baseline and for individuals at year 10.

The multivariable analysis shows nearly all factors to be significantly related to attrition except a few factors that are heavily related to other factors in the model. The results seen are similar between both the age-standardised and age-matched analyses. Individuals seen at year 10 differ from individuals at baseline in that they are better educated, have higher MMSE and have a family history of dementia. Individuals seen at the year 10 interview were more likely to have reported cardiovascular disease, depression, been a smoker or take alcohol. This analysis, whilst being age matched, is undertaken on two different overlapping birth cohorts, and smoking and drinking alcohol may well be factors that have cohort effects rather than attrition effects. A sensitivity analysis to missing-data response over the ten years of the interviews was undertaken and the results remained the same.

It is clear that cognition and education are the over-riding attrition effects seen consistently over the first two years and the next eight years as well as when investigating potential differences in individuals seen at 10 years compared at the same age seen just once. The same factors seen with attrition over a short time period are seen over a longer time period. No new factors appeared, though some effects increased in strength.

## Discussion

### Summary of results

Participants in MRC CFAS at year 10 differ from living, non-participants primarily by cognition with continuing participants having higher MMSE scores. After taking this into account, other more subtle effects are the increased likelihood of participation of people with a family history of dementia, and people with higher education.

Non-participants are composed of refusers and movers. Factors related to refusal in both the short term (first two years of the study) and longer term (the next eight were): poor cognition and <=9 years of education. Family history of dementia was also important in both periods though the previous analysis[9] did not investigate this. Increasing age was associated with refusal over the longer term (from year 2 to year 10), but was not associated with short term refusal. Other findings were that women were more likely to refuse, as were people with poorer self-reported health. Whilst most of the institutionalised died between waves, refusal was relatively low over the shorter term, but high over the longer term.

Factors related to moving in the shorter and longer term were not always the same. Movers have consistently been shown to be older and in worse health[1], and this was true for both short term and long term periods in CFAS. Individuals moving over the short term were more likely to have symptoms of dementia, ever had depression, and have ever smoked, and over the long term they were more likely not to have the top MMSE scores. The accommodation and marital status effect seen between baseline and two years was no longer apparent. This analysis probably is an indication of the relaxation of method due to small numbers rather than real differences between the time periods.

The increase of cardiovascular disease and depression is notable. These health questions are asked at each interview, with information on 'since we last saw you have you had a ...' it may well be that this recovers information from time prior to that anticipated by the question. This may indicate that self-report is more reliable with increasing interviews, as previous health episodes that were forgotten become remembered. Alternatively, individuals are who are ill are more likely to respond to surveys if they have previously been seen (e.g. in longitudinal surveys) rather than cross-sectional surveys or the survival of health factors has improved such that more individuals may be alive to report these conditions in old age.

### Critique

Analyses of attrition, even using multivariable analyses, will have thrown up many different 'significant' factors related to dropout. This will depend on, amongst other things, 1) what constitutes 'dropout', 2) how current are the time-varying factors, 3) how statistical models are chosen, 4) how characteristics are measured e.g. what instrument, and how categorised, 5) what factors are present in the model before other factors are tested, 6) sample size/power, and 7) peculiarities of the study design.

Reasons why people refused were only collected from year 6 onwards and hence it has not been possible to describe all refusers by reason for refusal. Broadly speaking, about 70% of refusals from year 6 onwards were 'active' (i.e. because the subject did not want to be interviewed), and about 30% were 'passive refusals' (because of the subject's poor health).

One possible explanation for finding an age effect associated with refusal from year 2 to year 10 is that the MMSE score at year 2 is not giving the same prediction for attrition eight years later as it is when measured just two years before. In the old, there is greater cognitive decline over time than in the young[19], and it is possible that the effect of age is acting as a surrogate for unmeasured cognitive decline.

The comparison of those followed up over ten years in comparison to the earlier birth cohort reveals differences that could be due to cohort effects, rather than attrition. A new study of the entire cohort will be needed to investigate which factors are cohort effects and which are attrition differences.

The size of the study means that relatively small effects (odds ratios of 1.2) are detectable.

### Findings in the context of the published literature

Less years of education have been reported to be associated with dropout in the US Longitudinal Study Of Aging [4]. Others have not found this to be related to refusal[20]. Cognition is consistently reported across studies[1]. This is the first time people with a family history of dementia have been reported to refuse less. This may indicate that such people are more willing to give their time and effort to interviews that can be seen as potentially contributing to new insights for a disease of importance to them personally.

As refusers in both periods tended to have roughly the same characteristics, studies may reasonably treat refusal at early waves as similar to refusal at later waves. It would be helpful to the interpretation of findings if more longitudinal studies provided careful analysis of dropout with appropriate adjustment of methods and results. Factors found to be related to dropout were still there after adjusting for cognition as measured by the MMSE and this finding has implications for many areas of investigation including disability.

## Conclusion

Many factors were found to be related to dropout in MRC CFAS before and after year 2. Refusers in both periods tended to have roughly the same characteristics suggesting studies can reasonably treat refusal at early waves as similar to refusal at later waves. Refusers tended to have poorer cognition, less years of education, did not have a family history of dementia and were women. Characteristics of people who moved differed between waves, but generally agreed with previous literature findings that the oldest and people in worse health move more. The use of the age-matched attrition over ten years indicated potential factors that may be better collected longitudinally compared to others where repeated cross-sectional studies may be preferable. These results will readily generalise to other studies, though more dedicated analysis of attrition would be welcomed and factors cannot be assumed to be stable across populations. In highly mobile societies such factors and adjustments will be increasingly important.

## Competing interests

The author(s) declare that they have no competing interests.

## Authors' contributions

All authors contributed to writing the paper. FM undertook the final analysis and is guarantor for the paper. MC undertook the original analysis. All authors have seen and agreed the final draft. MRC CFAS is a collaborative study and researchers are acknowledged using corporate authorship.

## Pre-publication history

The pre-publication history for this paper can be accessed here:



## Figures and Tables

**Table 1:** Number (%) of respondents by year 2 characteristics and year 10 status. Missing data excluded.

Group:	**Year 10 status**	**Interviewed†**		**Refused**		**Moved**		**Died**	
Total		3120		1373		342		3992	
Centre	Cambridgeshire	644	(21)	247	(18)	61	(18)	768	(19)
	Gwynedd	698	(22)	306	(22)	51	(15)	648	(16)
	Newcastle	575	(18)	232	(17)	66	(19)	834	(21)
	Nottingham	511	(16)	291	(21)	54	(16)	877	(22)
	Oxford	692	(22)	297	(22)	110	(32)	865	(22)
Women		1,887	(60)	900	(66)	233	(68)	2,211	(55)
[Age [Median (Interquartile range(IQR))]		71	(68–75)	72	(69–77)	72	(68–78)	78	(72–83)
Married/cohabiting		1,825	(59)	776	(57)	187	(55)	1,712	(43)
Residential/nursing home		15	()	24	(2)	5	(1)	410	(10)
Social class IV/V		492	(16)	275	(21)	33	(10)	759	(20)
Years of education <=9		1,711	(55)	855	(63)	182	(54)	2,532	(65)
Ever smoked		2,030	(65)	832	(61)	207	(61)	2,641	(68)
MMSE < 18		17	(1)	36	(3)	6	(2)	393	(10)
MMSE [Median (IQR)]		28	(26–29)	27	(24–28)	27	(24–28)	26	(22–28)
Townsend ADL 11+		130	(4)	98	(7)	317	(6)	2,672	(25)
ADL [Median (IQR)]		1	(0–3)	1	(0–4)	1	(0–3)	4	(1–11)
Fair/Poor self perceived health		620	(20)	353	(27)	62	(19)	1,392	(39)
Sight or hearing problem		583	(19)	280	(20)	80	(23)	1,277	(32)
Screening AGECAT O3+		26	(1)	47	(3)	6	(2)	516	(13)
Self reported depression		357	(12)	160	(12)	41	(12)	390	(11)
Chronic disease‡		1,894	(61)	847	(63)	211	(63)	2,818	(74)
Dementia prevalence		1	(0.5–2.1)	3.5	(1.8–6.6)	3.0	(0.9–9.5)	15.6	(13.6–18.6)
Depression prevalence		7	(0.5–10.0)	8.0	(0.5–11.9)	9.2	(4.1–19.1)	12.2	(10.0–15.0)
Family history of dementia		500	(16)	165	(12)	53	(16)	445	(12)
Disability	No ADL-IADL disability	2,491	(81)	986	(74)	261	(77)	1,770	(47)
	IADL disability only	432	(14)	219	(16)	56	(17)	768	(20)
	ADL disability	163	(5)	134	(10)	20	(6)	1,222	(33)
Alcohol	Don't drink	280	(10)	147	(12)	19	(6)	435	(12)
	Drink, not to excess	2,013	(71)	852	(69)	226	(74)	2,267	(65)
	Drink, excessive drinking	531	(19)	238	(19)	59	(19)	793	(23)
Cardiovascular diseases*									
	None	1,751	(57)	721	(54)	204	(60)	1,658	(45)
	One	919	(30)	391	(29)	86	(25)	1,217	(33)
	Two or more	426	(14)	215	(16)	48	(14)	800	(22)
Stroke		140	(5)	79	(6)	15	(4)	499	(14)

^†^There are 25 individuals who were interviewed at wave 4 who did not have wave 2 interviews ^‡ ^i.e. Parkinson's disease, angina, heart attack, stroke or arthritis. * i.e. Heart attack, hypertension – medication taken, angina, intermittent claudication

**Table 2:** Baseline characteristics of entire study and multivariable models for refusal and moving at year 2 and year 10

		**Baseline**		**Year 2**		**Year 10**		**Year 2**		**Year 10**	
Group:				**Refusers**		**Refusers**		**Moved**		**Moved**	
Total		13004		2520		1373		183		342	
				OR^†^	95% C.I.	OR^†^	95% C.I.	OR^†^	95% C.I.	OR^†^	95% C.I.

Age	65–69	3,184	(24)	1.0		1.0		1.0		1.0	
	70–74	3,150	(24)	1.0	(0.9–1.2)	1.1	(0.9–1.3)	0.9	(0.6–1.4)	0.9	(0.7–1.2)
	75–79	2,906	(22)	0.7	(0.6–0.8)	1.3	(1.1–1.6)	0.9	(0.6–1.4)	1.3	(1.0–1.8)
	80+	3,764	(29)	0.7	(0.6–0.8)	1.7	(1.3–2.1)	1.0	(0.6–1.5)	1.9	(1.4–2.7)
MMSE	Incomplete	428	(3)	8.2	(5.7–12.0)	4.0	(2.0–7.9)	2.4	(1.0–5.9)	1.2	(0.4–4.3)
	<21	1,546	(12)	7.6	(6.2–9.2)	1.7	(1.2–2.5)	1.5	(0.8–2.6)	1.4	(0.8–2.7)
	22–25	3,072	(24)	3.8	(3.2–4.4)	1.5	(1.2–1.8)	1.2	(0.8–1.8)	1.4	(1.0–2.1)
	26–28	4,955	(38)	1.5	(1.3–1.7)	1.2	(1.0–1.4)	0.8	(0.6–1.2)	1.5	(1.1–2.0)
	29–30	3,003	(23)	1.0		1.0		1.0		1.0	
Centre	Cambridgeshire	2,601	(20)	1.4	(1.2–1.6)	0.6	(0.5–0.8)	*0.8*	*(0.5–1.4)*	0.6	(0.4–0.8)
	Gwynedd	2,625	(20)	1.7	(1.4–1.9)	1.0	(0.8–1.3)	*1.2*	*(0.7–1.9)*	0.4	(0.3–0.6)
	Newcastle	2,524	(19)	1.3	(1.1–1.5)	1.0	(0.8–1.2)	*1.5*	*(1.0–2.3)*	0.7	(0.5–1.0)
	Nottingham	2,514	(19)	1.1	(0.9–1.3)	1.2	(1.0–1.5)	*1.2*	*(0.8–1.9)*	0.7	(0.5–0.9)
	Oxford	2,740	(21)	1.0		1.0		*1.0*		1.0	
Women		7,847	(60)	1.3	(1.2–1.5)	1.1	(1.0–1.3)	*0.8*	*(0.6–1.1)*	1.3	(1.0–1.7)
Years of education >9		4,657	(37)	0.8	(0.7–0.9)	0.8	(0.7–1.0)				
Type of home	Own home	11,424	(88)	1.0		1.0		1.0		*1.0*	
	Warden controlled	953	(7)	0.8	(0.7–1.0)	0.7	(0.5–1.0)	0.3	(0.1–0.8)	*0.9*	*(0.6–1.5)*
	Residential Home	593	(5)	0.5	(0.3–0.7)	3.0	(0.9–10.1)	0.9	(0.4–2.5)	*2.2*	*(0.6–7.3)*
Self perceived health	Poor	2,488	(20)	*1.1*	*(1.0–1.3)*	0.7	(0.6–0.9)				
	Fair	6,077	(48)	*1.0*		1.0					
	Good	3,335	(26)	*1.1*	*(0.9–1.2)*	1.1	(1.0–1.3)				
	Excellent	723	(6)	*1.2*	*(1.0–1.5)*	1.4	(0.9–2.1)				
Family history of dementia		1,512	(12)	*0.7*	*(0.6–0.8)*	0.8	(0.6–0.9)				
Never married/cohabiting		6,412	(50)					0.6	(0.5–0.9)	*1.1*	*(0.9–1.4)*
Screening AGECAT O3+		805	(6)					1.4	(0.6–3.3)	*0.9*	*(0.2–3.7)*
Self reported depression		1,122	(9)					1.7	(1.1–2.6)	*1.1*	*(0.7–1.5)*
Smoker		8,260	(65)					1.5	(1.1–2.2)	*0.9*	*(0.7–1.2)*

^†^Odds ratio (OR) and 95% confidence interval (C.I.) Factors from the previous or later waves models that were not in the best model are shown in italics.

**Table 3:** Characteristics at baseline and ten years for age-standardised group, adjusted odds ratios (and 95% confidence intervals) for age standardised and age matched analysis

Group:		**Baseline (n = 7013)**		**Year 10 (n = 3145)**		**Age standardised**		**Age matched**	
		**No**	**(%)**	**No**	**(%)**	**OR**^†^	**95% C.I.**	**OR**^†^	**95% C.I.**

Age (years)	70–74	366	(7)	32	(1)	7.2	(4.9–10.6)	Matched	
	75–79	2883	(40)	1238	(43)	1.0			
	80–84	2256	(31)	1016	(35)	0.8	(0.7–0.9)		
	85+	1508	(21)	859	(21)	0.7	(0.6–0.8)		
Centre	Cambridgeshire	1,385	(19)	645	(21)	0.8	(0.6–0.9)	Matched	
	Gwynedd	1,541	(21)	721	(23)	0.8	(0.7–0.9)		
	Newcastle	1,299	(19)	575	(18)	1.0	(0.8–1.1)		
	Nottingham	1,318	(18)	511	(16)	1.0	(0.9–1.2)		
	Oxford	1,470	(23)	693	(21)	1.0			
Women	Women	4,540	(65)	1,907	(60)	Not included		Not included	
Accomodation:	Own Home	5,737	(82)	2,693	(87)	1.0		Not included	
	Warden controlled	708	(10)	261	(8)	1.3	(1.1–1.6)		
	Residential home	537	(8)	184	(5)	1.0	(0.7–1.3)		
Marital status	Married/cohabiting	2,614	(39)	1,237	(41)	1.0		1.0	
	Single	622	(9)	227	(7)	1.3	(1.0–1.6)	1.4	(1.1–1.8)
	Widowed	3,458	(50)	1,547	(48)	1.2	(1.0–1.3)	1.2	(1.1–1.4)
	Divorced/Separated	154	(2)	109	(4)	0.6	(0.5–0.9)	0.6	(0.4–0.9)
Manual social class		3,758	(57)	1,543	(50)	Not included		Not included	
Years of education <=9		4,306	(64)	1,723	(55)	1.3	(1.2–1.5)	1.4	(1.2–1.6)
MMSE	Incomplete	218	(3)	41	(1)	5.6	(2.6–11.7)	6.8	(2.9–15.7)
	<= 21	630	(9)	189	(5)	2.9	(2.0–4.1)	2.9	(2.1–4.0)
	22–25	745	(10)	222	(7)	2.1	(1.7–2.6)	2.3	(1.8–2.8)
	26–28	2004	(29)	682	(21)	1.8	(1.6–2.0)	1.9	(1.7–2.2)
	29–30	3416	(49)	2011	(66)	1.0		1.0	
Screening AGECAT O3+		699	(10)	242	(7)	0.8	(0.6–1.0)	Not included	
Family history of dementia		687	(10)	505	(16)	0.6	(0.5–0.7)	0.6	(0.5–0.7)
Depression ever		443	(7)	447	(15)	0.4	(0.3–0.5)	0.4	(0.3–0.5)
Disability	No ADL-IADL disability	3,832	(56)	1,575	(54)	1.0		1.0	
	IADL disability only	1,192	(17)	762	(25)	0.6	(0.6–0.7)	0.6	(0.5–0.7)
	ADL disability	1,843	(26)	700	(21)	0.8	(0.7–0.9)	0.8	(0.7–1.0)
Townsend ADL 11+		1,269	(19)	608	(19)	Not included		Not included	
Stroke		595	(9)	309	(10)	Not included		Not included	
More than one cardiovascular disease		2,836	(42)	2,000	(66)	0.5	(0.5–0.6)	0.5	(0.5–0.5)
Self perceived health	Poor	1,216	(18)	551	(18)	0.9	(0.8–1.1)	1.0	(0.9–1.2)
	Fair	3,115	(47)	1557	(53)	1.0			1.0
	Good	1,954	(29)	747	(25)	1.5	(1.3–1.7)	1.3	(1.1–1.5)
	Excellent	382	(6)	117	(4)	1.9	(1.4–2.4)	1.7	(1.3–2.3)
Sight or hearing problem		2,773	(40)	1,020	(31)	1.6	(1.5–1.8)	1.6	(1.4–1.8)
Ever smoked		2,697	(60)	2,044	(66)	0.8	(0.7–0.9)	0.8	(0.7–0.9)
Alcohol	Don't drink	1,012	(17)	289	(10)	1.6	(1.3–1.8)	1.7	(1.4–2.1)
	Drink not to excess	4,016	(67)	2028	(71)	1.0		1.0	
	Drink evidence of excess	968	(16)	532	(19)	0.9	(0.8–1.1)	0.9	(0.8–1.1)

No: Number, % Percentages back-weighted for over sampling at baseline, ^†^Odds ratio (OR) and 95% confidence interval (C.I.)
